# Assessing the impact of storage conditions on RNA from human saliva and its application to the identification of mRNA biomarkers for asthma

**DOI:** 10.3389/fmolb.2024.1363897

**Published:** 2024-06-14

**Authors:** Poorna Manasa Bhamidimarri, David Fuentes, Laila Salameh, Bassam Mahboub, Rifat Hamoudi

**Affiliations:** ^1^ Research Institute of Medical and Health Sciences, College of Medicine, University of Sharjah, Sharjah, United Arab Emirates; ^2^ Rashid Hospital, Dubai, United Arab Emirates; ^3^ Department of Clinical Sciences, College of Medicine, University of Sharjah, Sharjah, United Arab Emirates; ^4^ Division of Surgery and Interventional Science, University College London, London, United Kingdom; ^5^ Centre of Excellence for Precision Medicine, Research Institute of Medical and Health Sciences, University of Sharjah, Sharjah, United Arab Emirates; ^6^ BIMAI-Lab, Biomedically Informed Artificial Intelligence Laboratory, University of Sharjah, Sharjah, United Arab Emirates

**Keywords:** salivary RNA, degradation, gene expression, diagnosis, RNA extraction

## Abstract

**Introduction:** Human saliva was used to develop non-invasive liquid biopsy biomarkers to establish saliva as an alternate to blood and plasma in translational research. The present study focused on understanding the impact of sample storage conditions on the extraction of RNA from saliva and the RNA yield, to be applied in clinical diagnosis. In this study, genes related to asthma were used to test the method developed.

**Methods:** Salivary RNA was extracted from three subjects using the Qiazol^®^ based method and quantified by both spectrophotometric (NanoDrop) and fluorometric (Qubit^®^) methods. RNA integrity was measured using a bioanalyzer. Quantitative PCR was used to monitor the impact of storage conditions on the expression of housekeeping genes: *GAPDH* and β-actin, and the asthma related genes: *POSTN* and *FBN2*. In addition, an independent cohort of 38 asthmatics and 10 healthy controls were used to validate the expression of *POSTN* and *FBN2* as mRNA salivary biomarkers.

**Results:** Approximately 2 µg of total RNA was obtained from the saliva stored at 40°C without any preservative for 2 weeks showing consistent gene expression with RNA stored at room temperature (RT) for 48 h with RNA*later*. Although saliva stored with RNA*later* showed a substantial increase in the yield (110 to 234 ng/μL), a similar Cq (15.6 ± 1.4) for the 18s rRNA gene from saliva without preservative showed that the RNA was stable enough. Gene expression analysis from the degraded RNA can be performed by designing the assay using a smaller fragment size spanning a single exon as described below in the case of the *POSTN* and *FBN2* genes in the asthma cohort.

**Conclusion:** This study showed that samples stored at room temperature up to a temperature of 40°C without any preservative for 2 weeks yielded relatively stable RNA. The methodology developed can be employed to transport samples from the point of collection to the laboratory, under non-stringent storage conditions enabling the execution of gene expression studies in a cost effective and efficient manner.

## 1 Introduction

Saliva has emerged as a promising tool to develop non-invasive diagnostic liquid biopsy methodologies and detect various diseases. In recent times, salivary biomarkers have been identified for oral (Lousada-Fernandez et al., 2018), colorectal (Loktionov, 2020), neck (Ovchinnikov et al., 2014; Chai et al., 2016; Wan et al., 2017), and gastric cancers (Li et al., 2018b). Discovery of biomarkers from saliva specimens has gained importance due to ease of collection and minimal sample processing time. Biomarkers known at present can be classified into a series of macromolecules such as proteins, RNA, and DNA (Fábryová and Celec, 2014; Loktionov, 2020). Saliva is a complex ultra-filtrated biofluid from plasma, containing enzymes, metabolites, and cell-free or extracellular RNA (exRNA) (Fábryová and Celec, 2014). The biochemical and physiological nature of saliva has extended the scope of salivary biomarker applications to help understand diseases that may not be directly related to the oral cavity (Loktionov, 2020).

The last two decades has seen an upsurge in the discovery of salivary biomarkers, mostly constituting protein or mRNA biomolecules (Dietz et al., 2012; Kishikawa, 2015; Maron, 2016), and at the same time, the advent of novel technologies in the molecular biology space has contributed to an overall simplification of the biomarker discovery process (Li et al., 2018a). A rise in the number of studies on circulating RNAs, non-coding small RNAs, and microRNA (Dietz et al., 2012; Kishikawa, 2015; Majem et al., 2015; Wan et al., 2017; Li et al., 2018b; 2018a) from saliva specimens has led to the identification of biomarkers related to cancer for molecular diagnostic purposes. In a recent study, DNA methylation patterns were analyzed from saliva samples collected from healthy and diseased patients where a significant difference was observed in the diseased specimens (Ovchinnikov et al., 2014). Based on various reports, saliva is now considered a reservoir of biomarkers for development of diagnostic and prognostic tools.

The major challenges in the identification of biomarkers include sample collection and isolation of the biomolecules to be tested. Many laboratories have optimized methods to isolate RNA from saliva and have proposed different conditions to obtain higher yield with greater stability (Dietz et al., 2012; Pandit et al., 2013; Mônica Ghislaine Oliveira et al., 2016; Madera Anaya and Suárez Causado, 2017; Sullivan et al., 2020). Several other studies have been performed to compare commercially available kits with the traditional QIAzol^®^ method (Pandit et al., 2013), usage of RNA-stabilizing agents, and the effect of sample collection procedures on the yield (Sullivan et al., 2020).

This study focuses on analyzing salivary biomarkers to understand disease progression in asthmatic patients. Asthma is a heterogeneous chronic complex disease of the airways (Wenzel, 2012). It has been studied for a long time to decode the complexity and identify specific biomarkers that differentiate each endotype (Wenzel, 2012). In the pursuit of salivary mRNA biomarkers differentiating non-severe and severe asthmatics from healthy controls, we encountered several challenges, especially during saliva sample collection and processing for RNA isolation. Current reports have addressed most of these challenges regarding isolation and yield; however, no study has yet been conducted to understand the effect of storage conditions and temperature on the samples collected and eventually on the overall yield of RNA for downstream molecular biology studies. In this study, we report on a cost-effective and simple method to process saliva samples for different biomolecular studies, which can be implemented even in low-resource settings with minimal equipment. In addition, preliminary analysis for the expression of asthma-related genes such as periostin (*POSTN*) and fibrillin 2 (*FBN2*) in the salivary RNA obtained from patient samples using the proposed method is presented.

## 2 Methodology

### 2.1 Sample collection and study design

The study was approved by the Ethics Committee of Dubai Health Authority and the University of Sharjah with REC (Research Ethics Committee) approval number DSREC-11/2017_04, and each subject provided written informed consent.

The study included three volunteers to assess the effect of storage conditions on salivary RNA. In addition, saliva from 20 mild/moderate (non-severe) asthmatics, 18 severe asthmatics, and 10 healthy individuals was similarly collected and stored until further use. The details of patient characteristics are provided in Supplementary Table S1.

**FIGURE 1:**
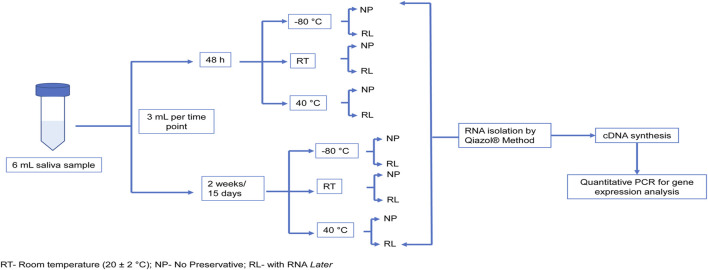
Schematic for the saliva sample processing to test the effect of storage conditions (temperature and duration) on RNA extraction. The initial sample collected from three healthy controls was distributed into different time points (48 h and 2 weeks) which were further distributed into temperatures −80°C, RT, and 40°C. The sample at each temperature was stored either with or without the preservative. All the samples were processed for extraction of RNA at the end of the time point.

Saliva samples from three healthy volunteers were collected according to the standard procedure. Briefly, participants were advised not to eat or drink for 2 hours before collection. Approximately 6 mL saliva was collected in a sterile tube by passive drooling after briefly rinsing the mouth with water. As the study was aimed to understand the effect of various storage time and temperatures, the saliva samples collected from each of the three subjects were distributed as per the schematic presented in Figure 1. The duration of storage was chosen as between 2 days (48 h) minimum and 2 weeks (15 days) maximum considering the minimum and maximum shipment/transfer times between the point of collection and the laboratory. In terms of the temperatures evaluated, −80 °C (common freezing storage temperature), RT (routine laboratory room temperature), and 40 °C (the maximum temperature a sample can be exposed on an average during transit) were chosen.

### 2.2 RNA isolation

The total RNA was isolated from saliva samples using the QIAzol^®^ method, as described earlier (Sullivan et al., 2020), with the following modifications; approximately 800 µL of the QIAzol^®^ lysis reagent was added for each 400 µL of the saliva sample, and the mixture was incubated for 5 min at room temperature (RT). One-fourth volume of chloroform was added to the lysed solution and incubated for 5 min at RT. The aqueous phase was separated by centrifugation at 14,000 *g* for 10 min at 4°C and aliquoted into an Eppendorf tube. The organic phase along with the lysed samples was stored on ice for DNA isolation. An equal volume of cold 2-propyl alcohol was added to the aqueous aliquot, and the RNA precipitate obtained was washed with ice-cold ethanol twice by centrifugation at 14,000 *g* for 10 min. The precipitate was air-dried and re-constituted with 30 µL of nuclease-free water (NFW).

### 2.3 RNA quantification

RNA was quantified using both a NanoDrop™ 2000 spectrophotometer (Thermo Scientific, United States) and a Qubit™ 4 fluorometer (Invitrogen, United States) using the Qubit™ RNA HS assay kit.

### 2.4 DNase treatment and cDNA synthesis

RNA was treated with DNase to remove any DNA contaminants by using the TURBO DNA-*free*™ kit (Invitrogen, United States) following the manufacturer’s instructions. The DNase-free RNA obtained was used to prepare cDNA using the high-capacity cDNA reverse transcription kit (Applied Biosystems, United States) which uses a mixture of oligo dT and random primers.

### 2.5 RNA integrity assessment

The RNA integrity number (RIN) was calculated using an automated electrophoresis instrument, the Agilent Bioanalyzer 2100 (Agilent Technologies, United States), using the Agilent RNA 6000 Nano Kit. One microliter of the RNA sample was separated on nanochip-based gel electrophoresis, and the RIN value was calculated based on the algorithm from the instrument, as described earlier (Schroeder et al., 2006). The lower the RIN value, the more RNA has degraded.

### 2.6 qPCR for housekeeping genes

Profiles of the housekeeping genes 18S rRNA (18S), β-actin (*ACTB*), and glyceraldehyde 3-phosphate dehydrogenase (*GAPDH*) were analyzed in the cDNA obtained from the different storage conditions. The primers were designed using the Primer3 tool (Untergasser et al., 2012), where each gene has two to three different sets of primers for different fragment lengths spanning one and two exons. Real-time PCR was performed using the Maxima SYBR green qPCR Master Mix (2X), and the quantification cycle (Cq) values were monitored for all of the primer sets (Table 1) by QuantStudio-3™ Real-Time PCR using the following conditions: hold stage 50°C for 2 min, 95°C for 10 min; 40 PCR cycles: 95°C for 15 s, 60°C for 1 min, 95°C for 15 s; melt curve stage: 60°C for 1 min, and 95°C for 1 s.

**TABLE 1:** List of the primer sequences used in the study.

Gene target	RefSeq ID	Primer name	Primer sequence 5′-3′	Amplicon fragment length (bp)	Number of exons the primer spans	Number of introns the primer spans
18S rRNA	NR_145820	18S sense (S)	TGA​CTC​AAC​ACG​GGA​AAC​C	114	--	---
		18S antisense (AS)	TCG​CTC​CAC​CAA​CTA​AGA​AC			
β-actin	NM_001101	ACTB1 S	CCAACCGCGAGAAGATGA	97	Two	One
		ACTB1 AS	CCA​GAG​GCG​TAC​AGG​GAT​AG			
		ACTB2 S	TCG​TGC​GTG​ACA​TTA​AGG​AG	109	One	Zero
		ACTB2 AS	TCA​GGC​AGC​TCG​TAG​CTC​TTT			
*GAPDH*	NM_001357943	GAPDH1 S	TTC​ATT​GAC​CTC​AAC​TAC​ATG	86	Two	One
		GAPDH1 AS	CCG​TTC​TCA​GCC​TTG​ACG​GTG			
		GAPDH2 S	TGT​CAG​TGG​TGG​ACC​TGA​CCT	148	One	Zero
		GAPDH2 AS	TCG​CTG​TTG​AAG​TCA​GAG​GAG			
*FBN2*	NM_001999	FBN2_1 S	CCG​GGG​AGA​ATG​ACG​AAA​AT	72	One	Zero
		FBN2_1 AS	TTC​AGG​AAT​GGT​TCC​GAT​GC			
		FBN2_2 S	TGA​TGA​ATG​TAT​GAT​AAT​GA	158	Two	One
		FBN2_2 AS	CAT​CAC​AGA​TAT​CAG​GAT​TG			
		FBN2_3 S	ATG​AAT​GCA​TCC​ACC​CCG​TT	182	One	Zero
		FBN2_3 AS	AAA​TGG​CCG​CCC​AGT​AAG​AA			
*POSTN*	NM_001135936	POSTN 1S	GGA​GGA​GCA​GTC​TTT​GAG​ACG			
		POSTN 1 AS	ATC​AGG​AAT​TAG​GAC​CTG​ATC​AAT	155	One	Zero
		POSTN 2 AS	AAT​TGG​GCC​ACA​AGA​TCC​GT	223	Two	One

### 2.7 qPCR for asthma-related genes

The cDNA obtained from the different storage conditions was analyzed for two important asthma-related genes: periostin (*POSTN*) and fibrillin 2 (*FBN2*). Primers for these genes were designed in Primer3 tool to check for different fragments spanning exons of variable length. The list of primers can be seen in Table 1.

Gene expression analysis for *POSTN* and *FBN2* was conducted on DNAse-treated salivary RNA isolated from 20 non-severe, 18 severe asthmatics, and 10 healthy controls using the primers POSTN_1 and FBN2_1 (Table 1), respectively, by quantitative PCR, as mentioned in Section 3.6. The Cq value of the gene of interest was normalized against the expression of the 18S rRNA gene from each sample (ΔCq), and the relative gene expression (2^−ΔΔCq^) was calculated using healthy controls (where ΔΔCq = ΔCq of asthmatic- ΔCq of healthy control).

### 2.8 Statistical analysis

Two-way ANOVA with non-repeated measures and Bonferroni post-test was used to analyze the data for RNA yield at different temperatures in the presence and absence of RNA*later*. The Mann–Whitney test was used to analyze the qPCR data comparing asthma patient samples with those from healthy individuals. *p* < 0.05 is considered statistically significant. All analyses were performed using GraphPad Prism software version 5.

## 3 Results

### 3.1 Storage at high temperatures without preservatives for longer times had a minimal effect on salivary RNA yield and quality

**FIGURE 2:**
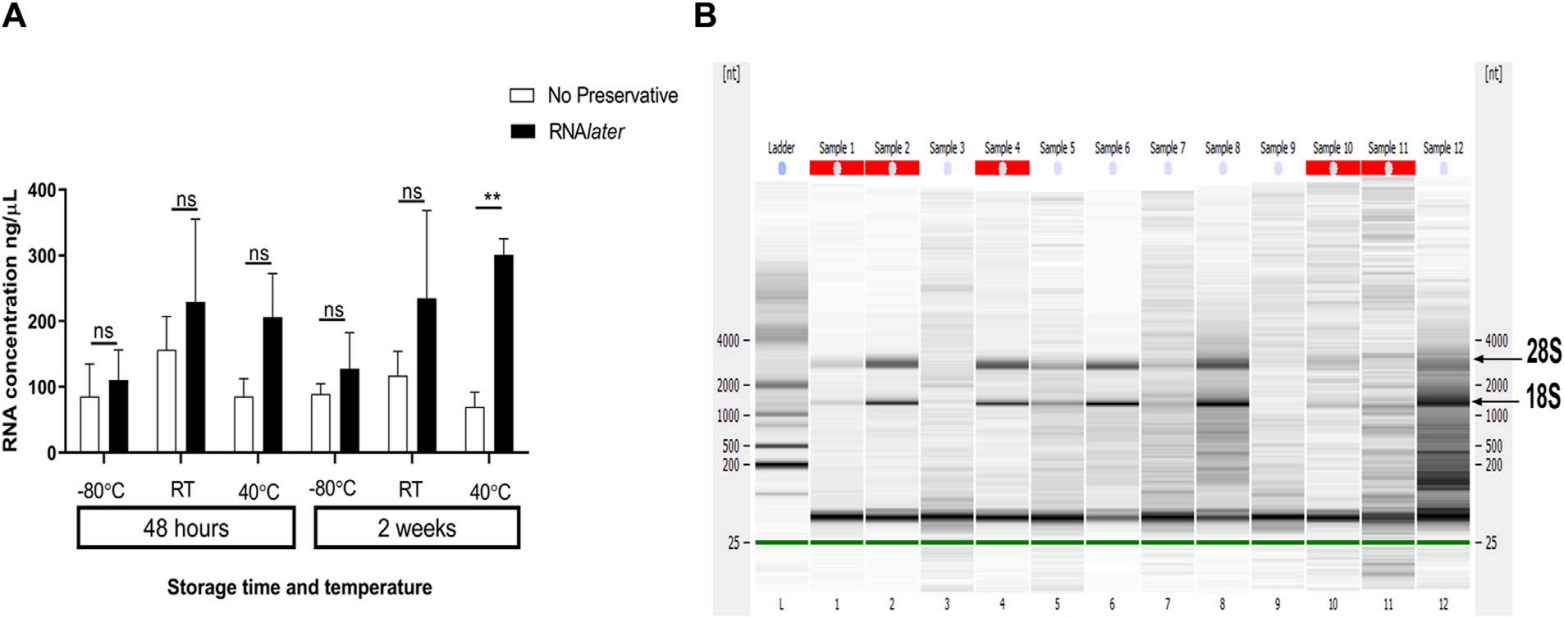
Effect of the storage conditions on RNA yield and integrity. Sample 1 through Sample 12 (Supplementary Table S4) is the Turbo DNase-treated RNA sample obtained from the saliva samples stored at RT, −80°C, and 40°C for 48 h and 2 weeks with/without RNA*later*. **(A)** Graph showing the RNA quantity obtained from the saliva samples stored at different temperatures and time points with/without the RNA stabilizing agent. ** signifies *p* < 0.01; ns, not significant. The details on the output from statistical analysis are provided in Supplementary Table S2. **(B)** Representative electropherogram obtained from Agilent Bioanalyzer for the RNA samples obtained from the saliva samples stored at different temperatures and time points with/without RNA*later*. The ladder size was mentioned as the number of the nucleotides. Sample ID and their corresponding description is provided in Supplementary Table S3. The bands corresponding to 28S rRNA and 18S rRNA were marked as 28S and 18S, respectively. The red dashes above the electropherogram indicate flags from the instrument and are displayed when the input RNA does not fit the standard total RNA profile.

The yield for the RNA obtained from three biological replicates ranged between 2.2 µg and 6.8 µg, and the A260/280 ratio was between 1.66 and 1.93 (Table 2). There was no significant difference between the RNA quantity for the saliva stored with RNA*later* or that stored without any preservative, irrespective of the temperature and duration of storage (Figure 2A). At a temperature of 40°C, saliva stored in RNA*later* for 2 weeks showed a significant improvement in yield (*p* < 0.01) when compared to saliva stored without preservatives at the same condition (Supplementary Table S2; Figure 2A).

**TABLE 2:** Quantity and quality of RNA obtained from the saliva sample stored at different temperatures and durations with/without the RNA*later*.

		Nanodrop ng/µL		A_260/280_		Qubit assay ng/µL		RIN	
Temperature		No preservative	RNA*later*	No preservative	RNA*later*	No preservative	RNA*later*	No preservative	RNA*later*
48 h	RT −80 °C 40°C	156.6 ± 50.6 85.2 ± 49.5 85.1 ± 27.2	229 ± 126.4 110.3 ± 46 206 ± 66.4	1.74 1.73 1.78	1.87 1.89 1.88	57.07 34.77 44.83	105.70 72.40 110.00	2.5 2.4 3.2	2.4 1.1 6.5
2 weeks	RT −80 °C 40°C	117.1 ± 37 89 ± 15.8 69.53 ± 22.4	234.8 ± 133.5 127.3 ± 55.1 216.3 ± 148.2	1.71 1.66 1.73	1.93 1.83 1.81	49.97 34.57 18.87	144.00 53.20 161	2.5 2.4 N/A	5.8 N/A 3.8

RT, room temperature (±20°C); RIN, RNA integrity number; N/A, not available.

RIN values measured using the Agilent Bioanalyzer ranged between 1.1 and 6.5, with higher values related to highly stable RNA (Figure 2B). Samples stored at 40°C for 2 weeks showed high degradation in the absence of preservatives; however, relatively stable RNA with RIN 3.8 was obtained at same condition in the presence of RNA*later* (Table 2; Figure 2B).

The total RNA quantity obtained from asthmatic patient samples using the QIAzol method ranged from 120 ng to 30 µg (Supplementary Table S3).

### 3.2 Analysis of expression of housekeeping genes using primers with variable amplicon sizes

**FIGURE 3:**
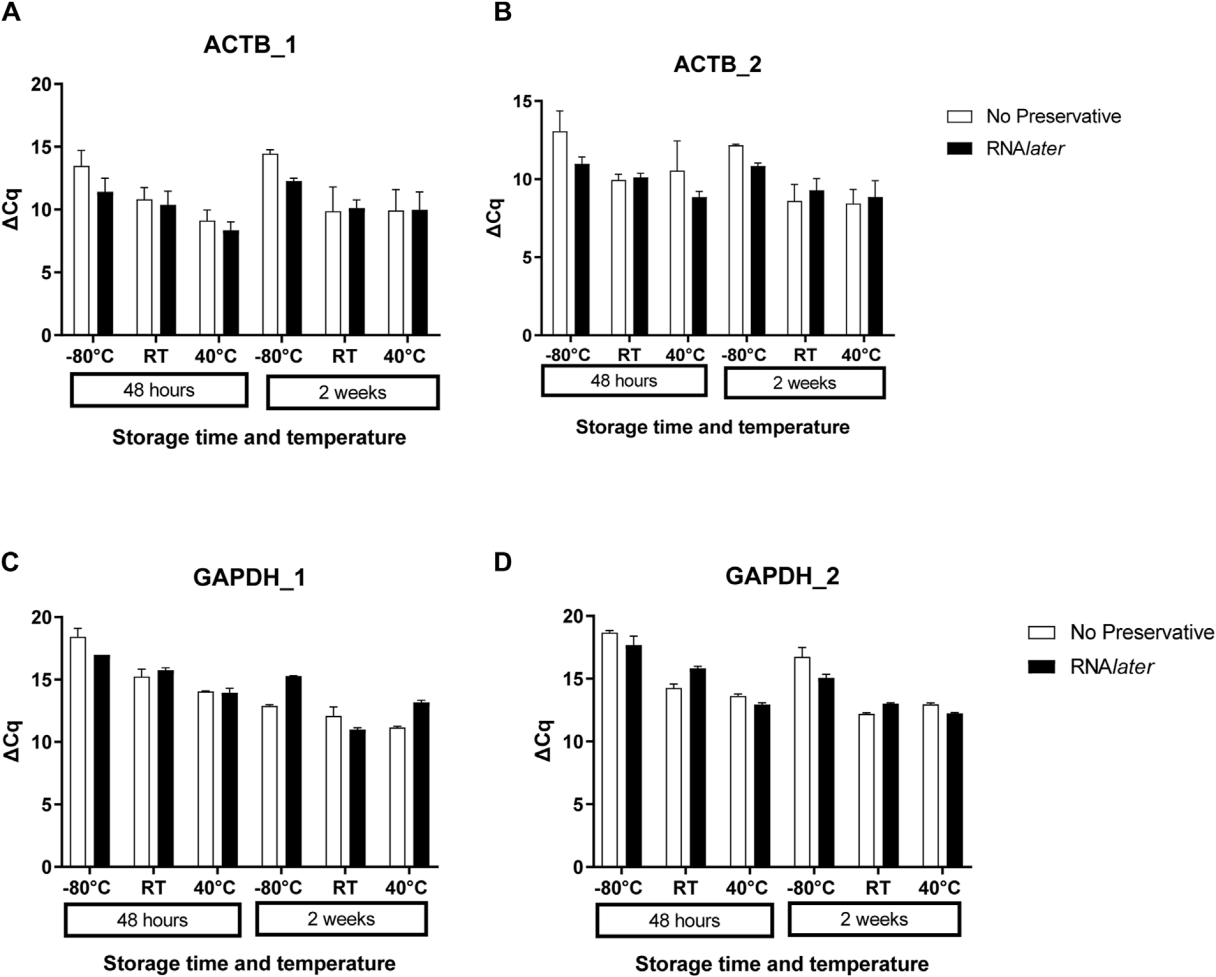
Expression analysis for housekeeping genes across the storage conditions among the three subjects. Bar plots for ΔCq values for housekeeping genes A and B. *ACTB* and C and D. *GAPDH* with variable amplicon sizes spanning single and double exons as shown in Table 1. ACTB1 **(A)** and ACTB2 **(B)** are less than 120-bp amplicons spanning two and single exons, respectively. GAPDH1 **(C)** and GAPDH2 **(D)** are less than 150-bp amplicons spanning two and single exons, respectively. In all the cases, there was no significant change noticed across the storage conditions.

Approximately 20 ng of cDNA obtained from reverse transcription PCR was used for quantitative real-time PCR for the primers designed for variable fragments spanning single exon and two exons of housekeeping genes β-actin and *GAPDH* (Table 1). The qPCR data revealed that there was no significant change in the Cq values for the 18S gene (15.6 ± 1.4) for all the samples stored at a higher temperature and for a longer duration without a stabilizing agent as compared to samples stored at RT and RNA*later* (Supplementary Figure S1). The same was noticed for other housekeeping genes *ACTB* and *GAPDH* in the case of both fragments spanning single and double exons (Figure 3). In both the cases, there was no significant change in ΔCq values at RT in the presence and absence of the preservative.

### 3.3 Analysis of asthma genes from the degraded RNA and validation in a patient cohort

The genes related to asthma *POSTN* and *FBN2* were analyzed by qPCR in the healthy control samples. The primers designed for fragments spanning more than one exon (POSTN_2 and FBN2_2) did not amplify across the samples, and the shorter fragments spanning a single exon (POSTN_1 and FBN2_1) gave consistent Cq values across the storage conditions (Supplementary Figure S2).

**FIGURE 4:**
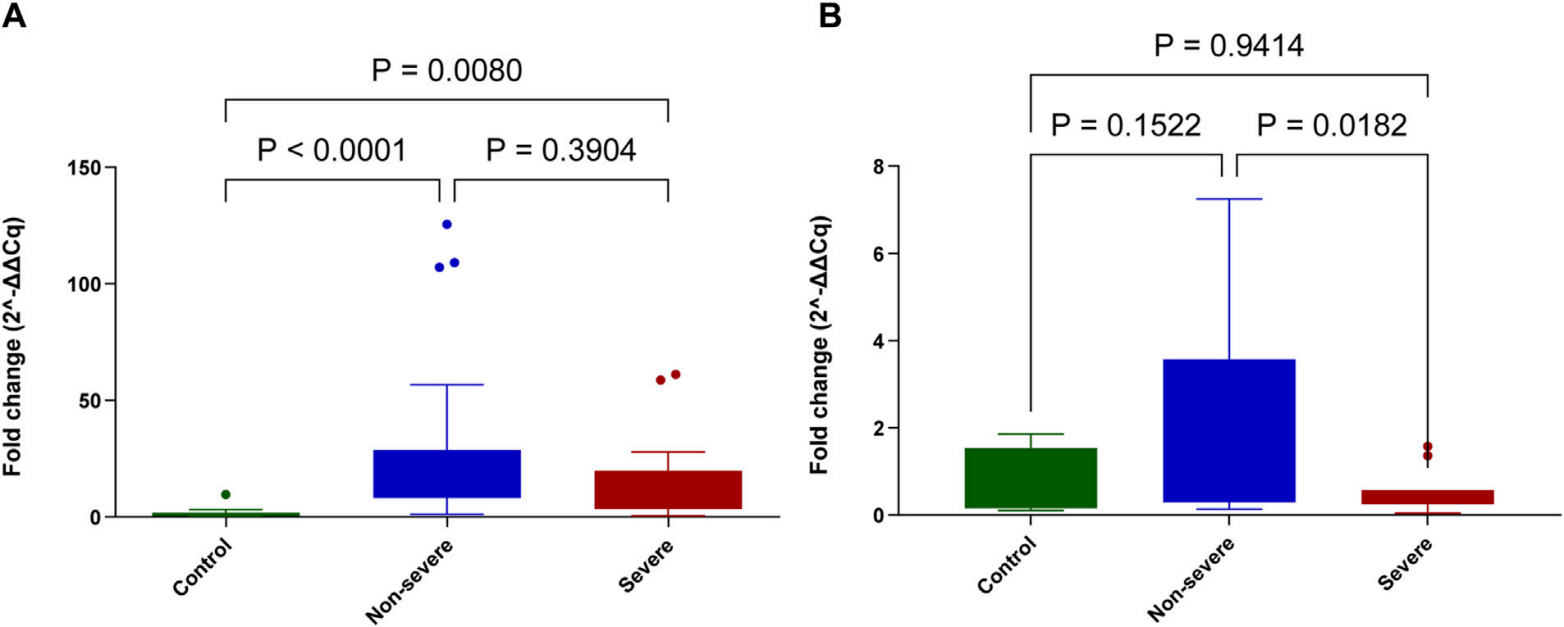
Differential gene expression for the genes related to asthma among 20 non-severe and 18 severe asthma patients’ saliva as compared to 10 healthy controls. **(A)** *FBN2* and **(B)** *POSTN*. The relative expression compared to healthy controls was presented as fold change (2^−ΔΔCq^). The Mann–Whitney test was conducted to test the significance between the groups. *p* < 0.05 was considered statistically significant.

The differential expression analysis for both genes *POSTN* and *FBN2* in the asthmatic samples (n = 38) as compared to 10 healthy controls (Figure 4) reflects the application of salivary RNA in gene expression analysis. *FBN2* showed a significantly higher expression among severe (*p* = 0.0080) and non-severe asthmatics (*p* < 0.0001) when compared to healthy controls. For *POSTN*, significantly increasing expression was observed among the non-severe group patients (*p* = 0.0182) when compared to severe asthmatics.

## 4 Discussion

Salivary biomarkers are important tools to diagnose the disease state of an individual in a non-invasive manner. The major challenges in the development of mRNA biomarkers for diagnosis are the stability and integrity of RNA extracted from patient specimens, which in turn depends upon the sample storage conditions. In this study, we have optimized a method to extract RNA from saliva stored at different temperatures and for different time durations. If the downstream molecular application is based on qRT-PCR and the primers are designed to span smaller fragment sizes, saliva stored either at RT or even at 40°C may yield optimal amounts of RNA necessary for gene expression assays.

Studies conducted so far have informed various methods of extraction and/or compared various kits for extraction; however, only limited studies were focused on storage conditions (Chiang et al., 2015). To our knowledge, this is the first study to address the effect of both temperature and duration of storage on salivary RNA yield. The results can be utilized to predict the requirements for clinical diagnostic labs wishing to use saliva samples in terms of collection, storage time, and temperature. Two studies by Sullivan et al. (2020) and Ostheim et al. (2020) focused on the challenges with degraded RNA and methods to improve the yields; however, in the present study, we have performed a comprehensive analysis of the effects in terms of both yield and degradation due to varied storage temperatures and timing.

RIN values ranging between 1.1 and 6.8 resulted in determining the gene expression for housekeeping genes with no significant variation among the Cq values. We recommend that the key factor is to design primers to span single or two exons with small amplicon size where the degraded RNA obtained did not show amplification for amplicon sizes spanning more than two exons and >180 bp. A previous study based on a RING trial between United States and United Kingdom recommended a similar workflow optimization to avoid pre-PCR variations in gene expression analysis, albeit for FFPE samples (Kapp et al., 2015).

Differential gene expression analysis among asthmatics and healthy controls was carried out to demonstrate the applicability of the method developed in the current study. Genes corresponding to asthmatic conditions identified earlier: *POSTN* (Li et al., 2015; Hachim et al., 2020) and *FBN2* (Choudhry et al., 2008; Giovannini-Chami et al., 2012) were chosen to evaluate the expression in salivary RNA collected from severe and moderate asthmatic patients in the UAE. *POSTN* is a well-known biomarker that was significantly expressed in higher quantities in non-severe asthmatics when compared to the severe asthma group. This biomarker is known to be a specific marker for Th2-type asthma; hence, further investigation on the asthma endotype of the samples collected might provide more specific results. However, in the present study, the focus was to assess the expression of biomarkers in degraded RNA for genes associated with asthma, demonstrating the effective application of the methodology developed in this study to clinical settings. Similarly, *FBN2*, a not so common asthma biomarker, was included and displayed a significantly higher expression in asthmatics when compared to healthy control samples. These data validate an optimized method for salivary RNA extraction and downstream processing for gene expression studies unlocking further avenues to carry out research and clinical studies using non-invasive techniques from saliva.

## 5 Conclusion

In conclusion, this study described the effect of storage temperatures ranging from room temperature to as high as 40 °C on the salivary RNA yield and integrity of the samples stored at two different time durations: 48 h and 2 weeks in the presence/absence of the stabilizing agent. Although the samples stored at higher temperatures for 2 weeks in the absence of a stabilizing agent yielded a lesser amount of highly degraded RNA when compared to the specimens stored in the stabilizing agent such as RNA*later*; the gene expression analysis for both the housekeeping and asthma-related genes using single exon spanning short fragment size primers indicated their suitability for clinical diagnosis. To the best of our knowledge, this is the first study that performs comprehensive analysis on the use of degraded salivary RNA in identifying mRNA liquid biopsy biomarkers for asthma. The methodology developed in this study can be used to support population screening and non-invasive diagnosis and prognosis of chronic complex diseases.

## Data Availability

The original contributions presented in the study are included in the article/Supplementary Material; further inquiries can be directed to the corresponding author.

## Supplementary material

SUPPLEMENTARY FIGURE S1 Graph displaying the Cq values for the 18S gene across all the storage conditions across the three biological replicates. No significant difference was observed across the conditions in Cq values. ns, not significant.

SUPPLEMENTARY FIGURE S2 ΔCq values for asthma-related genes **(A,B)**. *FBN2* and **(C)** *POSTN* were plotted for different fragment sizes (Table 1) and storage conditions where smaller fragments and single exon spanning fragments: FBN21 **(A)** and POSTN1 **(C)** gave consistent melt curves, and the fragments spanning two or more exons (data not shown) did not amplify.

## References

[B1] Chai R. C. Lim Y. Frazer I. H. Wan Y. Perry C. Jones L. (2016). A pilot study to compare the detection of HPV-16 biomarkers in salivary oral rinses with tumour p16INK4a expression in head and neck squamous cell carcinoma patients. BMC Cancer 16, 178. 10.1186/s12885-016-2217-1 26940728 PMC4778285

[B2] Chiang S. H. Thomas G. A. Liao W. Grogan T. Buck R. L. Fuentes L. (2015). RNAPro˙SAL: a device for rapid and standardized collection of saliva RNA and proteins. Biotechniques 58, 69–76. 10.2144/000114254 25652029 PMC4377224

[B3] Choudhry S. Taub M. Mei R. Rodriguez-Santana J. Rodriguez-Cintron W. Shriver M. D. (2008). Genome-wide screen for asthma in Puerto Ricans: evidence for association with 5q23 region. Hum. Genet. 123, 455–468. 10.1007/s00439-008-0495-7 18401594 PMC2664533

[B4] Dietz J. A. Johnson K. L. Wick H. C. Bianchi D. W. Maron J. L. (2012). Optimal techniques for mRNA extraction from neonatal salivary supernatant. Neonatology 101, 55–60. 10.1159/000328026 21791940 PMC3151004

[B5] Fábryová H. Celec P. (2014). On the origin and diagnostic use of salivary RNA. Oral Dis. 20, 146–152. 10.1111/odi.12098 23517132

[B6] Giovannini-Chami L. Marcet B. Moreilhon C. Chevalier B. Illie M. I. Lebrigand K. (2012). Distinct epithelial gene expression phenotypes in childhood respiratory allergy. Eur. Respir. J. 39, 1197–1205. 10.1183/09031936.00070511 22005912

[B7] Hachim M. Y. Elemam N. M. Ramakrishnan R. K. Hachim I. Y. Salameh L. Mahboub B. (2020). Confounding patient factors affecting the proper interpretation of the Periostin level as a biomarker in asthma development. J. Asthma Allergy 13, 23–37. 10.2147/JAA.S230892 32021310 PMC6955601

[B8] Kapp J. R. Diss T. Spicer J. Gandy M. Schrijver I. Jennings L. J. (2015). Variation in pre-PCR processing of FFPE samples leads to discrepancies in BRAF and EGFR mutation detection: a diagnostic RING trial. J. Clin. Pathol. 68, 111–118. 10.1136/jclinpath-2014-202644 25430497 PMC4316935

[B9] Kishikawa T. Otsuka M. Ohno M. Yoshikawa T. Takata A. Koike K. (2015). Circulating RNAs as new biomarkers for detecting pancreatic cancer. WJG 21, 8527–8540. 10.3748/wjg.v21.i28.8527 26229396 PMC4515835

[B10] Li F. Kaczor-Urbanowicz K. E. Sun J. Majem B. Lo H.-C. Kim Y. (2018a). Characterization of human salivary extracellular RNA by next-generation sequencing. Clin. Chem. 64, 1085–1095. 10.1373/clinchem.2017.285072 29685897 PMC7759158

[B11] Li F. Yoshizawa J. M. Kim K.-M. Kanjanapangka J. Grogan T. R. Wang X. (2018b). Discovery and validation of salivary extracellular RNA biomarkers for noninvasive detection of gastric cancer. Clin. Chem. 64, 1513–1521. 10.1373/clinchem.2018.290569 30097497 PMC7720197

[B12] Li W. Gao P. Zhi Y. Xu W. Wu Y. Yin J. (2015). Periostin: its role in asthma and its potential as a diagnostic or therapeutic target. Respir. Res. 16, 57. 10.1186/s12931-015-0218-2 25981515 PMC4437675

[B13] Loktionov A. (2020). Biomarkers for detecting colorectal cancer non-invasively: DNA, RNA or proteins? WJGO 12, 124–148. 10.4251/wjgo.v12.i2.124 32104546 PMC7031146

[B14] Lousada-Fernandez F. Rapado-Gonzalez O. Lopez-Cedrun J.-L. Lopez-Lopez R. Muinelo-Romay L. Suarez-Cunqueiro M. (2018). Liquid biopsy in oral cancer. IJMS 19, 1704. 10.3390/ijms19061704 29890622 PMC6032225

[B15] Madera Anaya M. V. Suárez Causado A. (2017). Evaluation of two RNA extraction methods in children’s saliva. Rev. Odontológica Mex. 21, e237–e243. 10.1016/j.rodmex.2018.01.014

[B16] Majem B. Rigau M. Reventós J. Wong D. (2015). Non-coding RNAs in saliva: emerging biomarkers for molecular diagnostics. IJMS 16, 8676–8698. 10.3390/ijms16048676 25898412 PMC4425103

[B17] Maron J. L. (2016). The neonatal salivary transcriptome. Cold Spring Harb. Perspect. Med. 6, a026369. 10.1101/cshperspect.a026369 PMC4772083 26684334

[B18] Mônica Ghislaine Oliveira A. Pérez-Sayáns M. Padín-Iruegas M.-E. Reboiras-López M. D. Suarez-Peñaranda J. M. López-López R. (2016). Comparison of RNA extraction methods for molecular analysis of oral cytology. Acta Stomatol. Croat. 50, 108–115. 10.15644/asc50/2/2 27789907 PMC5080564

[B19] Ostheim P. Tichý A. Sirák I. Davídková M. Stastna M. M. Kultova G. (2020). Overcoming challenges in human saliva gene expression measurements. Sci. Rep. 10, 11147. 10.1038/s41598-020-67825-6 32636420 PMC7341869

[B20] Ovchinnikov D. A. Wan Y. Coman W. B. Pandit P. Cooper-White J. J. Herman J. G. (2014). DNA methylation at the novel CpG sites in the promoter of MED15/PCQAP gene as a biomarker for head and neck cancers. Biomark. Insights 9, 53–60. 10.4137/BMI.S16199 25057238 PMC4085102

[B21] Pandit P. Cooper-White J. Punyadeera C. (2013). High-yield RNA-extraction method for saliva. Clin. Chem. 59, 1118–1122. 10.1373/clinchem.2012.197863 23564756

[B22] Schroeder A. Mueller O. Stocker S. Salowsky R. Leiber M. Gassmann M. (2006). The RIN: an RNA integrity number for assigning integrity values to RNA measurements. BMC Mol. Biol. 7, 3. 10.1186/1471-2199-7-3 16448564 PMC1413964

[B23] Sullivan R. Heavey S. Graham D. G. Wellman R. Khan S. Thrumurthy S. (2020). An optimised saliva collection method to produce high-yield, high-quality RNA for translational research. PLoS ONE 15, e0229791. 10.1371/journal.pone.0229791 32150588 PMC7062242

[B24] Untergasser A. Cutcutache I. Koressaar T. Ye J. Faircloth B. C. Remm M. (2012). Primer3—new capabilities and interfaces. Nucleic Acids Res. 40, e115. 10.1093/nar/gks596 22730293 PMC3424584

[B25] Wan Y. Vagenas D. Salazar C. Kenny L. Perry C. Calvopiña D. (2017). Salivary miRNA panel to detect HPV-positive and HPV-negative head and neck cancer patients. Oncotarget 8, 99990–100001. 10.18632/oncotarget.21725 29245955 PMC5725146

[B26] Wenzel S. E. (2012). Asthma phenotypes: the evolution from clinical to molecular approaches. Nat. Med. 18, 716–725. 10.1038/nm.2678 22561835

