# Supplementary material for: Assessing the impact of storage conditions on RNA from human saliva and its application to the identification of mRNA biomarkers for asthma
Source: Front Mol Biosci. 2024 Jun 14;11:1363897. doi: 10.3389/fmolb.2024.1363897 (PMC11211611; doi:10.3389/fmolb.2024.1363897)
Supplement: Supplementary file 1 [file Table1.DOCX]

**Supplementary Table 1.** Details on the patient characteristics

| **Name of the parameter** | **Non-severe asthma** | **Severe asthma** | **Healthy** |
| --- | --- | --- | --- |
| Number of Samples | 20 | 18 | 10 |
| Number of males | 4 | 8 | 4 |
| Number of females | 16 | 10 | 6 |
| Average age | 47 | 44.5 | 40.7 |
| Smokers | None | Four | one |
| FEV1 average (%) | 75.8 | 81.3 | 86.8 |
| Blood eosinophil average (Nx10^3^/µL) | 0.27 | 0.56 | 0.12 |
| Exacerbations (Avg) | 0.5 | 1.78 | - |
| Ig E levels (average) | 436 | 534 | 20 |
| Allergy (number) | 5 | 7 | 0 |

| **Time point & Temperature** | **No Preservative** | **RNA Later** | **Difference** | **95% CI of diff.** | **P value** |
| --- | --- | --- | --- | --- | --- |
| 48H -80°C | 85.2 | 110.3 | 25.1 | -132.7 to 182.9 | P > 0.05 (ns) |
| 48H RT | 156.6 | 229 | 72.4 | -85.36 to 230.2 | P > 0.05 (ns) |
| 48H 40 | 85.1 | 206 | 120.9 | -36.90 to 278.6 | P > 0.05 (ns) |
| W2 -80°C | 89 | 127.3 | 38.33 | -119.4 to 196.1 | P > 0.05 (ns) |
| W2 RT | 117.1 | 234.8 | 117.7 | -40.10 to 275.4 | P > 0.05 (ns) |
| W2 40 | 69.53 | 301.3 | 231.7 | 55.33 to 408.1 | P<0.01 (**) |

**Supplementary Table 2.** Summary table for two-way ANOVA for assessment of effect of preservative on RNA yield.

**Supplementary Table 3**. RNA concentration and quality details for asthmatic patient samples.

| **Sample ID** | **Asthma diagnosis** | **Conc. ng/µL** | **A260/280** | **Total Conc. (ng)** |
| --- | --- | --- | --- | --- |
| P1 | moderate | 108.9 | 1.78 | 3267 |
| P2 | moderate | 31.4 | 1.55 | 942 |
| P3 | severe | 101.9 | 1.82 | 3057 |
| P4 | severe | 21 | 1.91 | 630 |
| P5 | moderate | 99 | 1.57 | 2970 |
| P6 | moderate | 58.3 | 1.63 | 1749 |
| P7 | moderate | 4.1 | 1.74 | 123 |
| P8 | severe | 136.9 | 1.84 | 4107 |
| P9 | severe | 95.3 | 1.63 | 2859 |
| P10 | moderate | 63.6 | 1.49 | 1908 |
| P12 | moderate | 49.4 | 1.54 | 1482 |
| P13 | moderate | 71.4 | 1.51 | 2142 |
| P15 | moderate | 32.8 | 1.57 | 984 |
| P16 | moderate | 83.2 | 1.77 | 2496 |
| P17 | moderate | 155.1 | 1.61 | 4653 |
| P18 | moderate | 74.3 | 1.55 | 2229 |
| P24 | severe | 152.5 | 1.61 | 4575 |
| P26 | moderate | 211.7 | 1.68 | 6351 |
| P27 | severe | 768.5 | 1.94 | 23055 |
| P28 | severe | 318.6 | 1.81 | 9558 |
| P30 | moderate | 360.7 | 1.82 | 10821 |
| P31 | moderate | 503.8 | 1.79 | 15114 |
| P32 | severe | 1099.7 | 2.04 | 32991 |
| P33 | severe | 116.3 | 1.6 | 3489 |
| P34 | severe | 154.7 | 1.73 | 4641 |
| P35 | moderate | 307.8 | 1.79 | 9234 |
| P36 | severe | 203.6 | 1.68 | 6108 |
| P37 | moderate | 90.6 | 1.7 | 2718 |
| P38 | severe | 328.2 | 1.88 | 9846 |
| P39 | severe | 168.8 | 1.84 | 5064 |
| P40 | severe | 735.9 | 1.95 | 22077 |
| P41 | moderate | 134.7 | 1.68 | 4041 |
| P42 | severe | 89.5 | 1.75 | 2685 |
| P43 | moderate | 27.2 | 1.85 | 816 |
| P44 | severe | 146 | 1.66 | 4380 |
| P45 | severe | 145.6 | 1.62 | 4368 |
| P46 | moderate | 165.5 | 1.71 | 4965 |
| P49 | severe | 236.2 | 1.83 | 7086 |

**Supplementary Table 4**. List of samples and their codes

| Sample ID for Figure 2B | Storage temperature | Duration |
| --- | --- | --- |
| Sample 1 | RT; no preservative | 48 Hours |
| Sample 2 | RT; RNA*later* |  |
| Sample 3 | -80 °C; no preservative |  |
| Sample 4 | -80 °C; RNA*later* |  |
| Sample 5 | 40 °C; no preservative |  |
| Sample 6 | 40 °C; RNA*later* |  |
| Sample 7 | RT; no preservative | 2 weeks |
| Sample 8 | RT; RNA*later* |  |
| Sample 9 | -80 °C; no preservative |  |
| Sample 10 | -80 °C; RNA*later* |  |
| Sample 11 | 40 °C; no preservative |  |
| Sample 12 | 40 °C; RNA*later* |  |
