# Supplementary figures and images for: Assessing the impact of storage conditions on RNA from human saliva and its application to the identification of mRNA biomarkers for asthma
Source: Front Mol Biosci. 2024 Jun 14;11:1363897. doi: 10.3389/fmolb.2024.1363897 (PMC11211611; doi:10.3389/fmolb.2024.1363897)

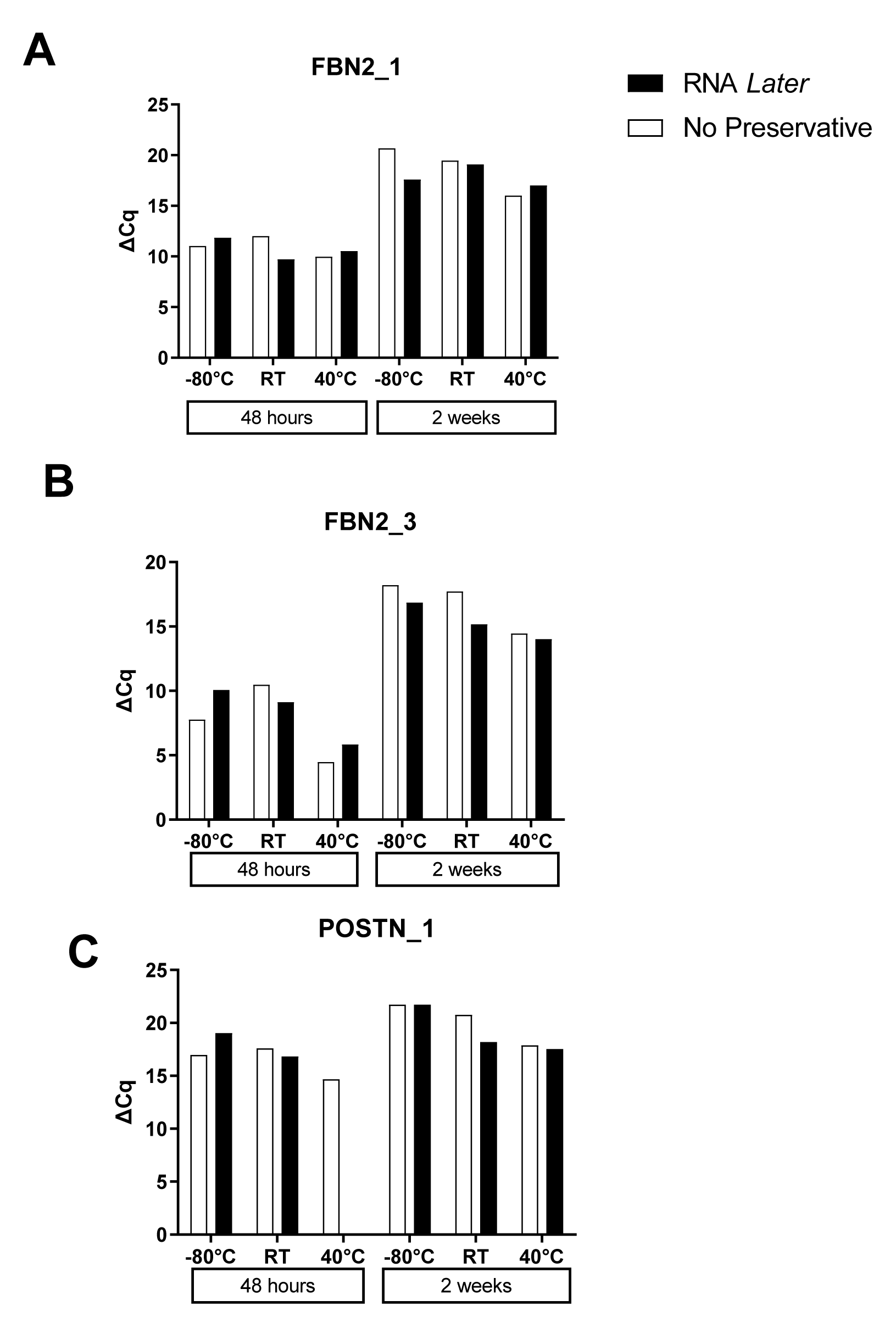

Supplement: Supplementary file 2 [file Image2.tif]

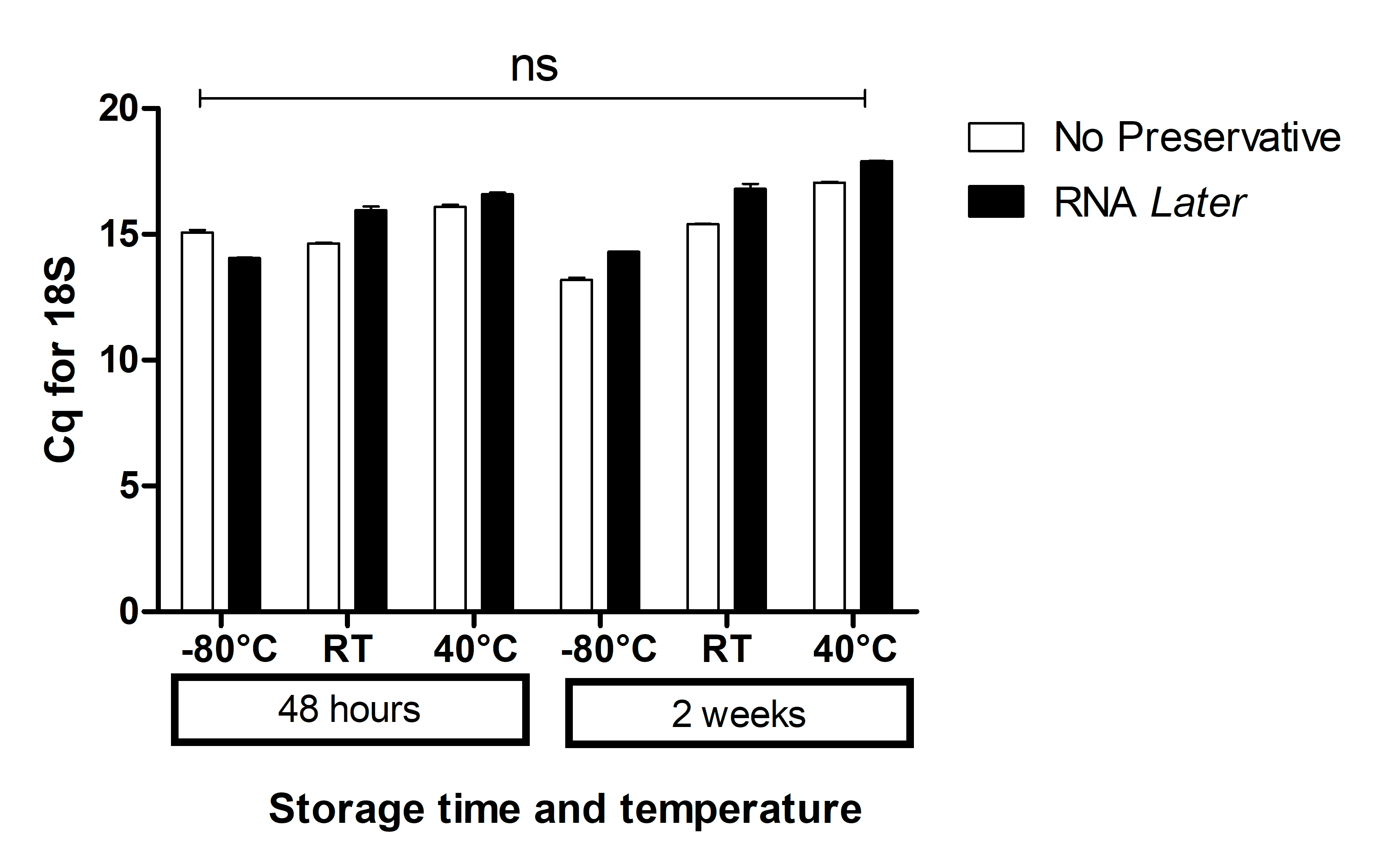

Supplement: Supplementary file 3 [file Image1.tif]
